# Effects of a cognitive dual task on variability and local dynamic stability in sustained repetitive arm movements using principal component analysis: a pilot study

**DOI:** 10.1007/s00221-018-5241-3

**Published:** 2018-03-27

**Authors:** Alessia Longo, Peter Federolf, Thomas Haid, Ruud Meulenbroek

**Affiliations:** 1 Donders Institute for Brain, Cognition and Behaviour, Radboud University, P.O. Box 9104, 6500 HE Nijmegen, The Netherlands; 2 Department of Sport Science, University of Innsbruck, Fürstenweg 185, 6020 Innsbruck, Austria

**Keywords:** Dual task, Largest Lyapunov exponent, Movement variability, Musculoskeletal disorders (MSDs), Postural reconfigurations, Principal component analysis (PCA)

## Abstract

In many daily jobs, repetitive arm movements are performed for extended periods of time under continuous cognitive demands. Even highly monotonous tasks exhibit an inherent motor variability and subtle fluctuations in movement stability. Variability and stability are different aspects of system dynamics, whose magnitude may be further affected by a cognitive load. Thus, the aim of the study was to explore and compare the effects of a cognitive dual task on the variability and local dynamic stability in a repetitive bimanual task. Thirteen healthy volunteers performed the repetitive motor task with and without a concurrent cognitive task of counting aloud backwards in multiples of three. Upper-body 3D kinematics were collected and postural reconfigurations—the variability related to the volunteer’s postural change—were determined through a principal component analysis-based procedure. Subsequently, the most salient component was selected for the analysis of (1) cycle-to-cycle spatial and temporal variability, and (2) local dynamic stability as reflected by the largest Lyapunov exponent. Finally, end-point variability was evaluated as a control measure. The dual cognitive task proved to increase the temporal variability and reduce the local dynamic stability, marginally decrease endpoint variability, and substantially lower the incidence of postural reconfigurations. Particularly, the latter effect is considered to be relevant for the prevention of work-related musculoskeletal disorders since reduced variability in sustained repetitive tasks might increase the risk of overuse injuries.

## Introduction

Movement variability is a pervasive and fundamental aspect of human performance. The redundancy of the motor system allows for the use of multiple strategies to perform any given task. Therefore, even highly monotonous tasks exhibit substantial variation over repetitions (Bernstein 1967). This inherent motor variability, which can manifest itself both in movements and in postures (Srinivasan and Mathiassen 2012), may be an important index of healthy and functional movements. In fact, movement variability has been considered a prerequisite for flexibility and adaptability, both crucial to motor learning (see, e.g., Wolpert et al. 2001; Dhawale et al. 2017), which may have implications for the prevention of overuse injuries (Hamill et al. 2012; Stergiou and Decker 2011). It has been suggested that one way to prevent overuse injuries or pain is to regularly alter the movement pattern in the execution of the repetitive task, thereby avoiding an overload of the same soft tissues (Bartlett et al. 2007).This hypothesis is of particular relevance in occupational contexts (Srinivasan and Mathiassen 2012; Madeleine et al. 2008; Fuller et al. 2011; Côté et al. 2005) for the prevention of work-related musculoskeletal disorders.

Characterizing movement variability remains an important challenge, since several different methods have been used to quantify movement variations, which not necessarily have the same meaning (Van Emmerik et al. 2016). A traditional way to quantify movement variability is to use discrete movement variables such as the standard deviation of movement amplitudes, i.e., spatial variability (Cignetti et al. 2009) or cycle durations, i.e., temporal variability (Danion et al. 2014). As opposed to traditional linear measures, the dynamical system theory (Kelso 1995) takes into account both spatial and temporal aspects of the movement and emphasizes notions such as stability and critical fluctuations to capture essential features of movements (Harbourne and Stergiou 2009), shifting the focus from isolated joints towards complex coordinated actions (Bartlett et al. 2007). The basic assumption of the dynamical system theory is that any multisegmental biological system which shows coordinated motor behavior by activity at the level of muscles, joints, and limbs, will find stable macroscopic coordination patterns by means of self-organization due to the intrinsic dynamics of the interactions at the microscopic level of its segments. The term stability in this context refers to the capacity of the system to counteract perturbations (Dingwell and Marin 2006). The largest Lyapunov exponent is a nonlinear measure used to determine the local aspects of stability (Segal et al. 2008; Dingwell et al. 2001; Hak et al. 2013). Local dynamic stability refers to the sensitivity of a system to small, intrinsic perturbations, and should not be confused with global stability. In fact, local fluctuations need to be attenuated to maintain global stability (Van Emmerik et al. 2016).

Variability and stability, although related, represent different concepts. Their exact relationship is not clear yet. On the one side, an increase in movement variability is considered a source of behavioral change in the system which signifies growing instability that may lead to a coordination shift to a different stable coordination pattern. On the other side, some behaviors which seem to be stable, may paradoxically show quite some variability (Dingwell and Marin 2006). Thus, it seems that variability does not always decrease when people get into or refine a stable behavioral state. In certain conditions, variability may actually increase. This contradictory relationship is noticeable when observing the rich behavioral repertoire of elite sport players or expert musicians (Harbourne and Stergiou 2009; Glazier et al. 2003).

An effective way to manipulate variability and stability in a cyclical motor task is adding a secondary cognitive task. This method is of particular interest in the context of the risks for work-related MSDs since cognitive demands are a relevant occupational factor which has been shown to affect sustained repetitive movements (Srinivasan and Mathiassen 2012; Bloemsaat et al. 2005). The underlying theory of studies on dual-tasking is that resources are limited, and they have to be shared between a cognitive and a motor task, consequently performance will suffer (Plummer and Eskes 2015). In dual-task paradigms, local dynamical stability might decrease, in terms of limited resources, since more difficult tasks demand more resources and as a consequence are less stable (Woollacott and Shumway-Cook 2002; Magnani et al. 2017). The effects of dual-task paradigms on movement variability, however, have mostly been controversial (Beurskens and Bock 2013; Beauchet et al. 2005).

In the current study, we designed a repetitive bimanual task with no postural constraints, which resembles real work-related environments. In the context of the just described views on movement variability and stability, we here propose an alternative analysis method. The approach consists of applying principal component analysis (PCA) on the subjects’ upper-body postural motion (Daffertshofer et al. 2004; Federolf et al. 2012) to isolate variability related to postural reconfigurations, i.e., intermittent and incidental changes in posture. We consider such postural configurations as non-linear transitions between two different (postural) coordination patterns. Our variability and stability analyses subsequently were directed to the most salient component following the PCA, which, in a sense, was not ‘contaminated’ by the non-linear, postural reconfigurations. Here, different variables were calculated (1) cycle-to-cycle spatial and temporal variability and (2) local dynamic stability as reflected by the largest Lyapunov exponent. As a control measure, endpoint variability was also assessed. In our view, exploring different methods to quantify movement variations at the level of the whole upper body may increase our understanding of the role of stability versus variability in sustained cyclical motion.

In summary, the goal of the current study was to investigate the effects of a concurrent cognitive task on different types of movement variability i.e., endpoint variability, postural reconfiguration, cycle-to-cycle variability, and on the local dynamic stability, i.e., largest Lyapunov exponent, in a sustained repetitive upper-extremity motor task. In line with the view that tasks that demand more resources are less stable, we hypothesized that the local dynamic stability, would decrease in the dual-task condition. With respect to movement variability, no particular effects were predicted because of contradictory or absence of earlier findings. Our pilot study was conducted to increase our understanding on how a cognitive load may contribute to an increased risk for work-related overuse injuries, a topic to which we will return in the “Discussion” section.

## Methods

### Participants

Thirteen right-handed healthy subjects (9 female, 4 male; 25.46 ± 3.46 years) volunteered for this study. No participant reported pain or history of injuries in neck, shoulder and arm regions. All participants gave informed written consent and the study was approved by an institutionalized ethics review board.

### Procedure

**Fig. 1:**
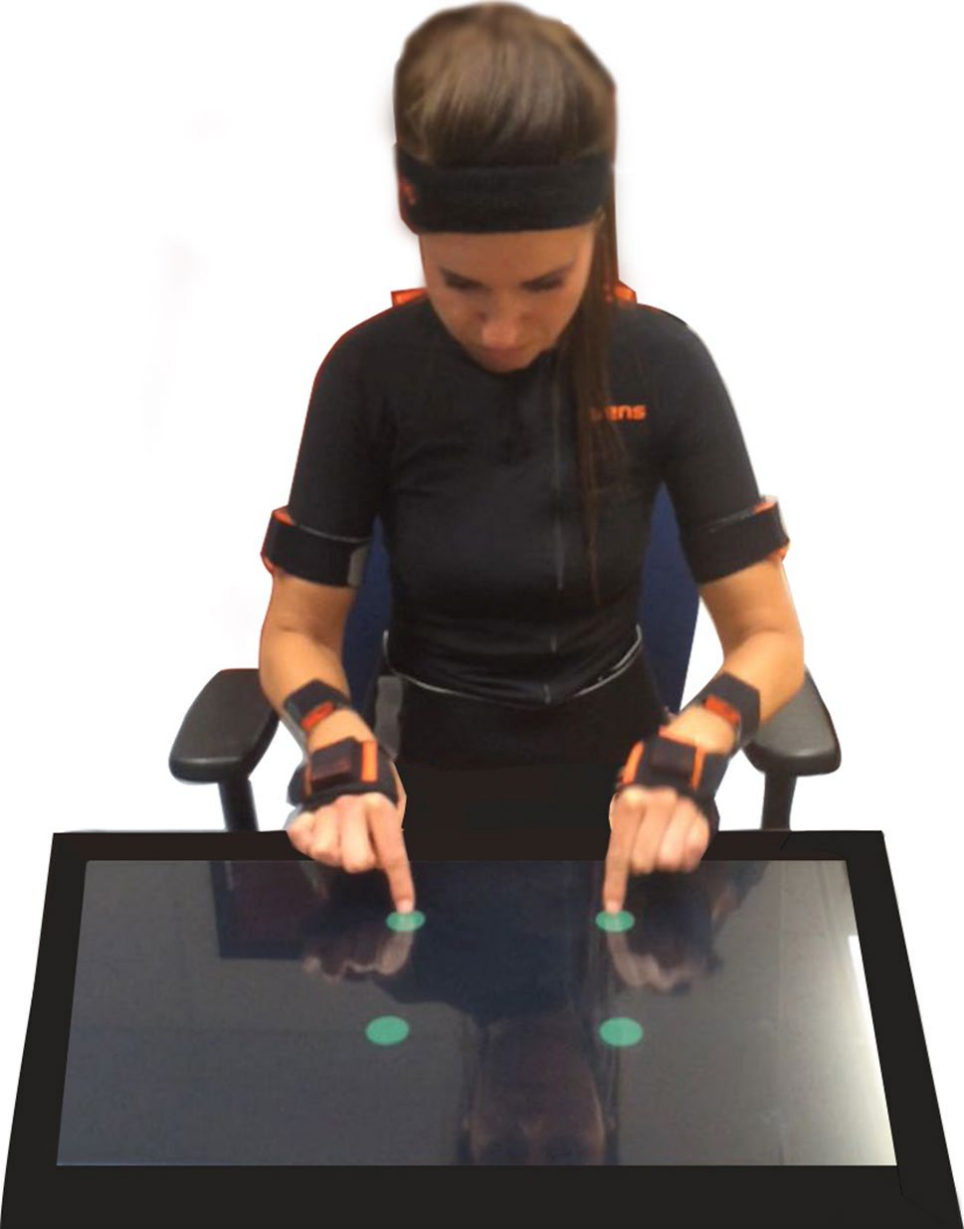
Experimental setup

The protocol consisted of two trials of 5 min each, whose order was counterbalanced between participants. In one trial, a motor task was performed solo (*M*) and in the other a motor task was performed in combination with a cognitive task (*M* + *C*). In the cognitive task, participants counted aloud backwards in multiples of three. In the motor task, participants performed a sustained repetitive task on a multi-touch screen, tapping two pairs of visually presented targets with both hands simultaneously and in-phase (Fig. 1). Participants could perform the task freely (without specified rhythm or posture), with the only requirement of touching the targets as fast and as accurate as possible. The motor task shares common features with the bimanual Fitts’ task used in previous studies (Longo and Meulenbroek 2018; Shea et al. 2012; Amazeen et al. 2005), however, no specific task variations as regards movement amplitude and target width, were applied in the current study to enhance its monotony. Before starting the measurements, participants were asked to adjust the chair height and distance to the touch screen to find the most comfortable position. The configuration of the chair was then maintained in all trials. All participants performed a warming-up trial for a minimum of 30 s or until they felt comfortable with the task.

### Equipment

A custom-made script in Python 2.7 2010 (Python Software Foundation, Beaverton, OR, USA) was implemented to present the stimuli and record the endpoint position on a 27′′ touch screen (1920 × 1080 resolution; ProLiteb Iiyama, Iiyama Corporation, Tokyo, Japan). Four targets of 27 mm in diameter were presented, with a between-targets distance of 125 mm in anterior direction and a distance of 155 mm between the targets of the two hands. Xsens MVN BIOMECH motion capture suit (Xsens technologies BV, Enschede, The Netherlands) was used to record upper body kinematics at 60 Hz. Eleven sensor units were placed on the head, sternum, pelvis, shoulders, upper arms, forearms and hands following the recommendations by Xsens. Anatomical measurements and calibrations were performed according to the procedures provided by Xsens. Data acquisition was done via the accompanying software (MVN Studio 4.2, Xsens technologies BV, Enschede, Netherlands) which calculates the kinematic data. Thirty joint angles: the 3D angle configurations of wrist (2: left and right), elbow (2), shoulder (2) and four column angles: C1–Head, T1–C7, T9–T8, L5–S1, were considered for further analysis. All joint angles were expressed in local coordinate systems following the guidelines of the International Society of Biomechanics (Wu et al. 2005).

### Data analysis

Touch screen data (XY coordinates) of the fingertip positions realized during the 5-min task were used to quantify the endpoint variability. We used the standard measure of variable error (VE), which was defined as the mean distance of all movement endpoints to the mean endpoint (Gordon et al. 1994). For further calculations, we determined for each participant the mean of VE for all four targets (VE_*m*_). One subject was excluded from this analysis since data were missing.

Xsens data were used to investigate other types of variability and local dynamic stability, which were the primary interest in the present study. For this purpose, a PCA was applied using the 30 joint angles as 30-dimensional input vectors. Prior to data analysis, the first 5 s were excluded from the raw dataset, to avoid analyzing settling-in behavior. For each trial, every angle vector was normalized by subtracting the trial-mean. Then, a single input matrix was created with the normalized vectors as columns and the data of all subjects and both conditions (*M, M* + *C*) concatenated vertically. Finally, a single PCA was calculated on this combined input matrix to facilitate direct comparisons between participants (Federolf 2016; Gløersen et al. 2017). The first three principal components (PCs) were considered for the analysis of postural reconfigurations which reflect the movement variability related to the volunteer’s postural changes ( see also Longo et al. 2018). The first principal component was further examined for the analysis of cycle-to-cycle variability and local dynamic stability in repetitive cycles. All calculations in the current study were implemented in Matlab R2015a (MathWorks Inc., Natic, MA, USA).

### Postural reconfigurations

**Fig. 2:**
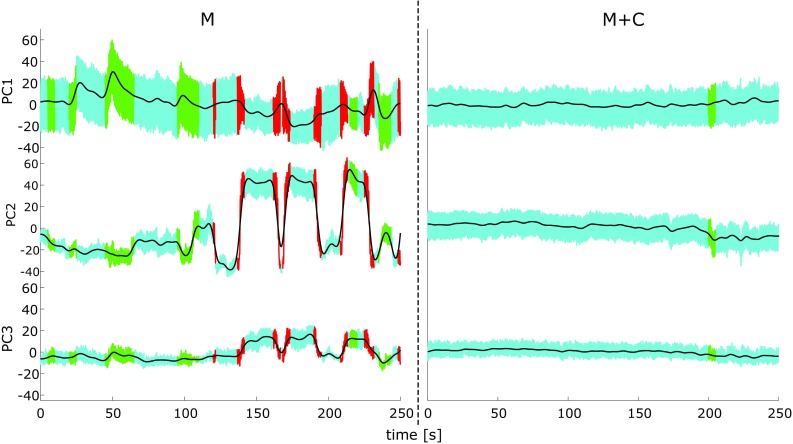
Representative dataset of a 5 min trial of the motor (*M*) and the motor + cognitive (*M* + *C*) trial of one arbitrarily selected volunteer: the first three PCs are shown. The tapping movement between two pairs of targets is printed as a colored line, respectively, quasi-stationary phases (cyan), non-stationary phases (green), and transitions (red). The black line represents the low pass-filtered underlying trend

Changes in the postural configuration (Fig. 2) were determined by first defining the trends of the first three PCs (black lines) through a low pass filter (Butterworth filter; cut-off 0.1 Hz). Then, the trends were used to classify four phases: events—interruptions or unusual movements during the execution of the task; transitions—rapid changes from a postural configuration to another; non-stationary phases—gradual changes between postural configurations; quasi-stationary phases—unchanging postural configurations. In particular, events were defined by subtracting each trend from its PC; an event was marked if the deviation of the PC time series from its trend was lower than half its total average (i.e., pause within the repetitive task), or if it exceeded two times its total average (e.g., unusual movement). The slope of the trends underlying the residual time periods were used to delineate transitions, non-stationary, and quasi-stationary phases, respectively. Specifically, transitions were defined if the absolute value of the slope for a minimum of 100 samples exceeded a threshold of 0.1 and non-stationary phases if the absolute value of the slope for a minimum of 300 samples exceeded a threshold of 0.02. Thresholds and number of samples used were specific for our setup and best identified the four phases. Time periods that were not allocated to any of the former phases were marked as quasi-stationary phases. If a criterion for any of the phases was met in one PC, then this period was delineated accordingly in all PC time series (Fig. 2). For further comparisons, the cumulated duration per minute of each phase (*D*) was calculated. Thus, for each condition (*M, M* + *C*), four dependent variables were defined: *D*_e_ (events), *D*_t_ (transitions), *D*_ns_ (non-stationary phases), and *D*_qs_ (quasi-stationary phases).

### Cycle-to-cycle variability

**Fig. 3:**
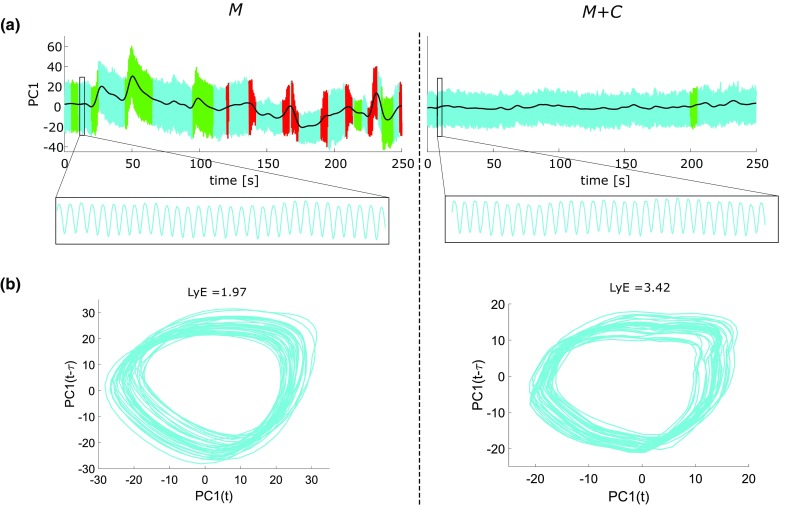
**a** Representation of PC1 of the motor (*M*) and the motor + cognitive (*M* + *C*) trial of one arbitrary selected subject. The enlargement shows 30 cycles selected in the quasi-stationary phases for the analysis of cycle-to-cycle variability. **b** State space representation of 30 cycles of the same representative subject for the analysis of the largest Lyapunov exponent

Thirty consecutive cycles were selected in the PC1 time series in a quasi-stationary phase (Fig. 3a). The cycles selected corresponded to the first quasi-stationary phase of at least 30 cycles, i.e., longest consecutive cycles that could be detected among all participants and both conditions in the relevant time periods. A cycle was defined as a back and forward movement, starting from the targets closer to the body. The starting points of the cycles corresponded to the local maxima of PC1. Spatial (SD_*C*_) and temporal (SD_*T*_) variability were calculated on the 30 selected cycles. SD_C_ was calculated by first interpolating each cycle such that it was represented by 100 samples (i.e., expressed in percent). For each sample, the standard deviation between cycles was determined. Finally, the mean of the standard deviations over the whole cycle was calculated. SD_*T*_ was assessed as the standard deviation of the movement duration between cycles. The mean of the movement duration (*T*_*m*_) between the 30-selected cycles was also assessed as a control measure.

### Local dynamic stability

The largest Lyapunov exponent (LyE) was calculated for the same 30 cycles selected in the PC1-time series. LyE is a measure of local dynamic stability, which quantifies the exponential rate of separation of neighboring trajectories of the attractor. LyE was calculated by first constructing a state space representation of the time series (Fig. 3b). The time delay (τ) was determined using the average mutual information (AMI; Fraser and Swinney 1986 )and the embedding dimension (*m*) using a false nearest neighbor algorithm (Kantz 1994) Therefore, *m* = 2 and τ = 9 were selected. Finally, LyE values were calculated for the time series using Wolf’s algorithm (Wolf et al. 1985; Buzzi et al. 2003.)

### Statistical analysis

To determine changes in the postural configuration, the cumulated duration per minute of each phase (*D*_e_, *D*_t_, *D*_ns_, *D*_qs_) was compared between the two conditions (*M, M* + *C*). As the data were not normally distributed, a Wilcoxon signed-rank test was used. Variables *D*_e_, *D*_t_, *D*_ns_ are independent and were analyzed applying a Šidák correction for multiple comparisons, thus reducing the *α*-level for statistical significance to *α* = 0.0174. For completeness, also variable *D*_qs_, which directly depends on the other variables (*D*_qs_ = 60 s − [*D*_e_ + *D*_t_ + *D*_ns_]) was analyzed; also applying the corrected *α*-threshold of *α* = 0.0174. For the analysis of cycle-to-cycle variability, local dynamic stability and endpoint variability the data were normally distributed, therefore, a paired-samples *t* test was used to compare SD_*C*,_ SD_*T*_, *T*_*m*_, LyE, and VE_*m*_ for both conditions. Here, the *α*-level for statistical significance was set to *α* = 0.05. Statistical analyses were performed using SPSS Version 22 (IBM, Chicago, IL, USA).

## Results

### Results of the principal component analysis

Principal components 1–3 represented 44.9, 16.9, and 9.9% of the overall variance in the kinematic data, respectively. Figure 2 shows an example of the first three PC score time series of the *M* and *M* + *C* trial for one selected subject. The first principal component (PC1) represented the movement component containing the largest variance and, in the current study, PC1 was dominated by the cyclic movement pattern of the task. PC2 and PC3 represent variance orthogonal to PC1 and were largely affected by postural reconfigurations of the subjects.

### Motor task versus motor + cognitive task

**Fig. 4:**
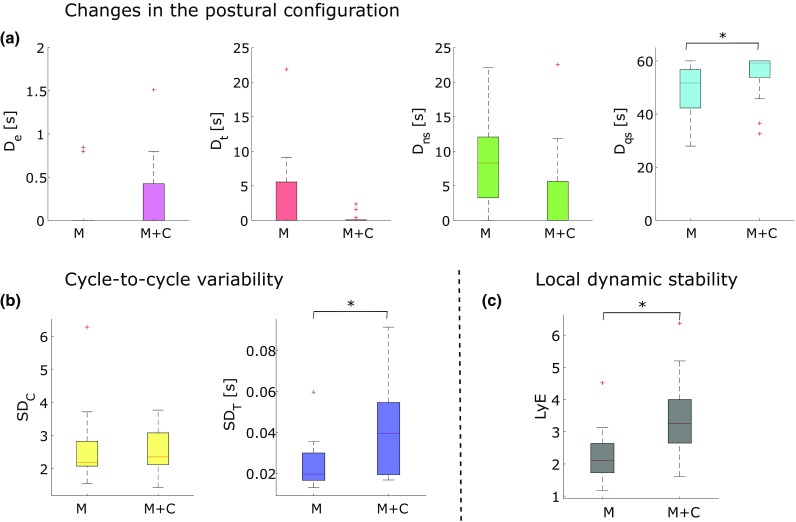
**a** Box plots of cumulative duration per minute of events (*D*_e_; magenta), transitions (*D*_t_; red), non-stationary phases (*D*_ns_; green) and quasi-stationary phases (*D*_qs_; cyan)for the motor (*M*) and the motor + cognitive (*M* + *C*) trial; **b** box plots of spatial variability (SD_*C*_) and temporal variability (SD_*T*_) for *M* and *M* + *C*; **c** box plot of the largest Lyapunov exponent (LyE) for *M* and *M* + *C*. Significant between condition effects are indicated by an asterisk

With respect to endpoint variability, a statistical trend was observed in VE_*m*_ which decreased in the *M* + *C* [9.34 (± 3.22) mm] compared to the *M* [11.01 (± 3.78) mm] trials [*t*(11) = 1.83, *P* = 0.095, *d* = 0.48]. Postural reconfigurations (Fig. 4a) revealed a significant main effect in *D*_qs_ which was higher in the *M* + *C* than in the *M* trial (*Z* = 2.43, *P* = 0.015, *r* = 0.67). A statistical trend was found in *D*_t_ (*Z* = 2.02, *P* = 0.043, *r* = 0.56), indicating more frequent changes in the *M* than in the *M* + *C* trial. No significant differences between conditions were found in *D*_ns_ (*Z* = 1.73, *P* = 0.084, *r* = 0.48) and in *D*_e_ (*Z* = 0.67, *P* = 0.5, *r* = 0.19).

Cycle-to-cycle variability (Fig. 4b) revealed a significant main effect between conditions in SD_*T*_ [*t*(12) = 2.39, *P* = 0.036, *d* = 0.75] indicating higher temporal variability in the *M* + *C* than in the *M* trial. However, no significant differences between conditions were observed in SD_*C*_ [*t*(12) = 0.45, *P* = 0.664, *d* = 0.16] and in *T*_*m*_ [*t*(12) = 1.24, *P* = 0.239, *d* = 0.33]. Local dynamical stability (Fig. 4c) decreased in the *M* + *C* as compared to the *M* trial as reflected by an increase of LyE [*t*(12) = 2.36, *P* = 0.036, *d* = 0.6].

## Discussion

The current pilot study explored the effects of a concurrent cognitive task on different types of movement variability, i.e., endpoint variability, postural reconfiguration, cycle-to-cycle variability, and on the local dynamic stability, i.e., largest Lyapunov exponent, in a sustained repetitive upper-extremity motor task. In agreement with our hypothesis and the view that tasks that demand more resources are less stable, the local dynamic stability decreased under dual-task conditions (Fig. 4c). However, the effects of the secondary cognitive load on different types of movement variability revealed contrasting results as compared to earlier studies that used different variability measures (Beauchet et al. 2005; Hollman et al. 2007). Temporal variability increased (Fig. 4b), suggesting that the cognitive task caused interference due to the competition for attentional resources necessary for the motor task. The increase of temporal variability with an additional cognitive load is in line with dual-task interference effects reported earlier (Dubost et al. 2006; Beauchet et al. 2005). Spatial variability was not affected by the counting task and the endpoint variability marginally decreased. Simultaneously, the incidence of postural reconfigurations significantly decreased in the dual-task condition (Fig.4a), indicating that participants adopted fixed postures for longer periods of time. Since motor variability has been purported as beneficial for avoiding overuse injuries and pain (Bartlett et al. 2007; Srinivasan and Mathiassen 2012), the decrease in postural readjustments due to dual tasking may constitute a risk factor for MSDs.

The postural readjustment results may be interpreted from the viewpoint of dynamical systems theory as follows. Generally, in dual-task paradigms, the challenge for a motor system performing movements and a cognitive task is to adapt to the secondary task demands without reducing the quality of movement performance. The main purpose of a dynamical system then is to reach or maintain global stability. Goal-directed actions are supported by reducing the number of biomechanical degrees of freedom of the motor system through the formation of functional synergies affording preferred and stable coordination patterns. However, a stable system does permit flexible and adaptive motor behavior, encouraging free exploration of coordination changes to be able to acquire different stable motor solutions over time, a mechanism known to enhance motor learning (Glazier et al. 2003). Now, if the motor system is perturbed due to a concurrent cognitive task, as we observed in the current study, local stability may be reduced. A dynamical movement system can try to attenuate local fluctuations and maintain a stable coordination pattern by adopting another functional solution or coordination mode that suits the dual-task constraints better. The result of this process may be that the system is constrained at the joint level thus reducing the incidence of postural reconfigurations.

A novelty of the present study is the application of PCA for the assessment of different types of variability and local-dynamic stability. Using this approach, we moved away from quantyfing the variability of isolated joints by a limited number of pre-selected kinematic variables, and instead, moved towards metrics such as postural reconfigurations of the whole upper body which allowed us to capture complex multijoint coordination and thus provide a fuller account of multijoint cyclical movements while coping with a cognitive load. Further, we attempted to better understand what distinct parameters measuring variability and stability reflect in sustained upper-extremity motion. Our results show that LyE and temporal variability reflect unwanted fluctuations in performance due to reduced control with an increase in task difficulty.The incidence of postural reconfigurations, however, reflects a potential beneficial variability due to the dynamics of the human movement system. Another benefit of distinguishing between different types of variability by means of PCA is that the LyE can then be calculated on quasi-stationary phases. Stationarity of the underling time series is a prerequisite for this calculation, but in human movement studies this stationarity is often difficult to define. However, one limitation of this approach was that the number of consecutive cycles needed for the calculation of LyE was limited by the occurrence of postural reconfigurations, nonetheless the length selected is considered adequate for the analysis (cf. Wolf et al. 1985). Further, due to the novelty of the current approach and the low sample size, our findings need to be taken with caution.

In conclusion, the current findings suggest that under cognitive demands, the temporal variability and dynamic instability of cyclical arm movements increase. Simultaneously, at the postural level, cognitive loads led to a decreased incidence of postural reconfigurations. Particularly, for the prevention of MSDs, this reduced postural variability should be carefully monitored since postural reconfigurations may play a role in the prevention of overuse injuries.
